# Brain activations during bimodal dual tasks depend on the nature and combination of component tasks

**DOI:** 10.3389/fnhum.2015.00102

**Published:** 2015-02-26

**Authors:** Emma Salo, Teemu Rinne, Oili Salonen, Kimmo Alho

**Affiliations:** ^1^Division of Cognitive Psychology and Neuropsychology, Institute of Behavioural Sciences, University of HelsinkiHelsinki, Finland; ^2^Advanced Magnetic Imaging Centre, Aalto University School of Science and TechnologyEspoo, Finland; ^3^Helsinki Medical Imaging Center, Helsinki University Central HospitalHelsinki, Finland; ^4^Helsinki Collegium for Advanced Studies, University of HelsinkiHelsinki, Finland; ^5^Swedish Collegium for Advanced StudyUppsala, Sweden

**Keywords:** dual task, divided attention, fMRI, phonological processing, spatial processing

## Abstract

We used functional magnetic resonance imaging to investigate brain activations during nine different dual tasks in which the participants were required to simultaneously attend to concurrent streams of spoken syllables and written letters. They performed a phonological, spatial or “simple” (speaker-gender or font-shade) discrimination task within each modality. We expected to find activations associated specifically with dual tasking especially in the frontal and parietal cortices. However, no brain areas showed systematic dual task enhancements common for all dual tasks. Further analysis revealed that dual tasks including component tasks that were according to Baddeley's model “modality atypical,” that is, the auditory spatial task or the visual phonological task, were not associated with enhanced frontal activity. In contrast, for other dual tasks, activity specifically associated with dual tasking was found in the left or bilateral frontal cortices. Enhanced activation in parietal areas, however, appeared not to be specifically associated with dual tasking *per se*, but rather with intermodal attention switching. We also expected effects of dual tasking in left frontal supramodal phonological processing areas when both component tasks required phonological processing and in right parietal supramodal spatial processing areas when both tasks required spatial processing. However, no such effects were found during these dual tasks compared with their component tasks performed separately. Taken together, the current results indicate that activations during dual tasks depend in a complex manner on specific demands of component tasks.

## Introduction

Performing two or more cognitive tasks simultaneously is assumed to require executive functions such as coordination of cognitive resources (Alvarez and Emory, 2006). It has been proposed that brain activity during dual tasks that cannot be associated with either of the component tasks would reflect such functions. Previous functional magnetic resonance imaging (fMRI) studies suggest that dual tasking would activate prefrontal cortical areas involved in coordination of limited processing resources (Corbetta and Shulman, 2002; Schubert and Szameitat, 2003; Johnson and Zatorre, 2006; Stelzel et al., 2006; Johnson et al., 2007) and posterior parietal cortical areas involved in control (e.g., shifting) of attention (Corbetta and Shulman, 2002; Shomstein and Yantis, 2004; Corbetta et al., 2008).

However, performing two cognitively demanding tasks simultaneously may deteriorate performance in either task or in both tasks. It is generally assumed that dual task performance deteriorates when the component tasks require the same limited sensory, cognitive or cortical resources (Welford, 1952; Mowbray, 1953; Pashler, 1994; Roland and Zilles, 1998; Alais et al., 2006). Furthermore, due to limitations in dividing attention between two sensory modalities, task related activity in the auditory and visual cortex is lower during intermodal divided attention than during auditory or visual selective attention, respectively (Johnson and Zatorre, 2006). Taken together, these results suggest that dual tasking comprises several processes that are not yet fully understood.

Previous studies have used a limited number of task combinations (e.g., two unimodal single tasks and one bimodal dual task) in order to identify activations associated specifically with dual tasking. Therefore, in the present study, we examined the effects of three auditory and three visual component tasks and their nine combinations on brain activity during dual tasking. The dual tasks comprised an auditory phonological, spatial or simple (speaker-gender) discrimination task and a visual phonological, spatial or simple (font-shade) discrimination task. All tasks were performed on identical stimuli and required identical motor responses to targets. The auditory and visual phonological tasks (A_Phon_ and V_Phon_, respectively), as well as the auditory and visual spatial tasks (A_Spat_ and V_Spat_, respectively), were designed to be as similar as possible to each other in terms of task requirements. The auditory and visual simple tasks (A_Simp_ and V_Simp_, respectively), in turn, were designed to require modality specific processing (voice and luminance contrast discrimination, respectively). This design allowed us to study the functional significance of activations associated with different dual tasks. Our previous study (Salo et al., 2013), using identical stimuli and the same participants as the present study, investigated activations associated with the three auditory component tasks and the three visual component tasks when performed as the only task. To evaluate whether all dual tasks activate some common brain areas, we compared the present dual task data with single task data from our previous study.

We expected that especially dual tasks requiring parallel phonological or spatial processing would show strong activation modulations. Our previous study showed that, when performed separately, the A_Phon_ and V_Phon_ tasks activate the same area in the left prefrontal cortex involved in phonological processing and that the A_Spat_ and V_Spat_ tasks activate the same area in the right inferior parietal cortex involved in spatial processing (Salo et al., 2013). Thus, we expected to find strong modulation of activity especially in these areas. Finally, we hypothesized that all dual tasks would show activity enhancements in the same areas of dorsolateral prefrontal cortex and posterior parietal cortex involved in task coordination and control of attention, in addition to some activity decrements in the primary sensory cortices due to intermodally divided attentional resources.

## Methods

### Participants

Participants (*N* = 15, 8 female) were native Finnish speakers, between 20 and 35 years of age (mean 25 years). All participants were right handed, had normal hearing, normal or corrected-to-normal vision, and no history of psychiatric or neurological illnesses (all self-reported). An informed written consent was obtained from each participant before the experiment. The experimental protocol was approved by the Ethical Committee of the Hospital District of Helsinki and Uusimaa, Finland. One to three weeks before the present dual task session, all participants had participated in an fMRI session where all component tasks of the present study were performed separately in single task conditions (Salo et al., 2013). In addition, 1–7 days prior the present session the participants took part in a short practice session to familiarize them with dual task instructions.

### Stimuli

The exact stimulus parameters are reported in our previous study (Salo et al., 2013). In brief, auditory stimuli consisted of 17 meaningless consonant-vowel and vowel-consonant syllables each having a duration of 250 ms. Seven syllables started with a vowel (ab, ad, ag, ah, ak, ap, at) and 10 started with a consonant (du, fu, ku, lu, mu, nu, pu, ru, su, vu). The syllables were uttered by four female and four male native Finnish speakers. Interaural time difference was used to produce eight spatial locations organized in two spatial categories: central (four locations, ca. 2.5° or 5° to the left or right from midline) and peripheral (four locations, ca. 20° or 25° to the left or right from midline). Visual stimuli (duration 250 ms) consisted of 17 consonant letters (height ca. 0.018°). Participants were required to discriminate between letters with a *name* starting with a vowel and letters with a *name* starting with a consonant (e.g., in English, the name of letter R is pronounced like “are” and thus starts with a vowel, while the name of letter T is pronounced like “tea” and starts with a consonant). The Finnish names of seven chosen consonant letters started with a vowel and ended in a consonant (F, L, M, N, R, S, X) and names of 10 chosen consonant letters started with a consonant and ended in a vowel (B, C, D, G, H, J, K, P, T, V). The letters were presented on a gray background (Red, *R* = 128, Green, *G* = 128, Blue, *B* = 128) in either darker gray (four shades) or a lighter gray (four shades). Moreover, they occurred in eight locations either centrally near the fixation asterisk (four diagonal locations ca. 0.029° from fixation) or more peripherally (four diagonal locations ca. 0.075° from fixation). Asynchronous auditory and visual sequences were presented in bimodal 30 s blocks that alternated with 15.3 s breaks. Within each modality, stimulus onset-to-onset intervals varied randomly between 375 and 625 ms in 10 ms steps (rectangular distribution).

### Procedure

**Figure 1:**
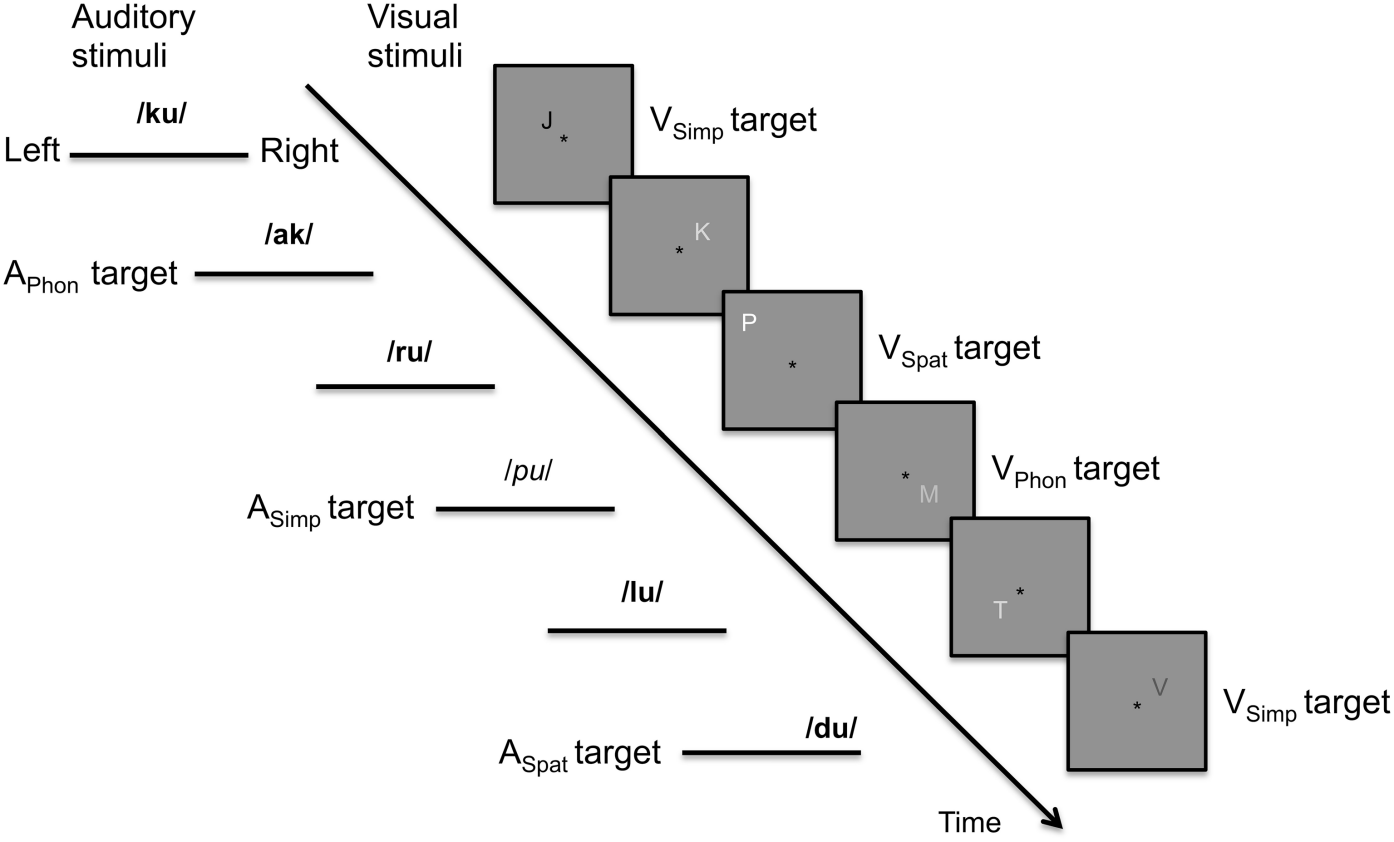
**Schematic illustration of the auditory and visual stimulus streams in the present study**. In the auditory stream, the targets in phonological, spatial, and simple tasks were syllables starting (vs. ending) with a vowel, presented at the left or right lateral (vs. central) loci or spoken by a female (vs. male) voices. In the figure, female and male voices are indicated in italic and bold font, respectively. In the visual stream, the targets in phonological, spatial, and simple tasks were letters with their name beginning (vs. ending) with a vowel, letters presented farther off (vs. closer to) the fixation asterisk or letters written with a font darker (vs. lighter) than the background.

The participants were presented with concurrent asynchronous streams of spoken syllables and written letters that varied in their phonological, spatial and modality specific (voice or font shade) features (Figure 1). For both modalities, there were three different component tasks. In the auditory phonological, spatial and simple tasks (A_Phon_, A_Spat_, and A_Simp_, respectively), targets were syllables starting with a vowel, syllables presented at more peripheral (left or right) locations, and syllables uttered by a female speaker, respectively. In the visual phonological, spatial and simple tasks (V_Phon_, V_Spat_, and V_Simp_, respectively), targets were letters with their name beginning with a vowel, letters at more peripheral locations, and letters presented with a darker gray than the background, respectively. The component tasks were combined to make nine bimodal dual tasks (A_Phon_V_Phon_, A_Phon_V_Spat_, A_Phon_V_Simp_, A_Spat_V_Phon_, A_Spat_V_Spat_, A_Spat_V_Simp_, A_Simp_V_Phon_, A_Simp_V_Spat_, and A_Simp_V_Simp_). During all dual tasks, the participants were required to focus on a black fixation asterisk constantly shown at the center of the screen and to press a button with their right index finger to the auditory and visual targets as fast as possible. During the breaks, they focused on the fixation asterisk and waited for the next task. Eye position was monitored with an iView X MEyetrack LR long range camera and a matching iView X MEyetrack mirror box (SensoMotoric Instruments, Teltow, Germany).

An instruction chart (including the fixation asterisk) was shown in the middle of the screen for 5 s before the onset of the next block. The chart consisted of two rows and four columns of text (in Finnish). The upper and lower rows of the first column contained a black letter A (for Auditory tasks) and V (for Visual tasks), respectively. The rows of the second column contained “female voice” and “dark letter.” The third column had “vowel beginning” and the fourth column “peripheral” on both rows. The columns of the chart were identical for all dual task combinations, except that the target feature for each modality was indicated with black letters, the text in black on the first row indicating target feature in the auditory modality and the text in black on the second row indicating target feature in the visual modality. The other texts were written in gray letters that were darker than the background.

For each of the nine dual task combinations, there were seven blocks and thus altogether 63 blocks were presented. All participants were presented with the same series of 63 stimulus blocks. However, the order of the tasks to be performed in these blocks was randomized separately for each participant. Each block contained 60–80 auditory and 60–80 visual stimuli with a target probability of 0.2 per modality. In both auditory and visual stimulus sequences, stimulus features (17 syllables or letters, 8 auditory and 8 visual locations, 8 font shades and 8 voices, 4 male and 4 female) varied randomly, except that any feature that was used as a target in one of the tasks (i.e., syllables starting with a vowel, peripheral auditory location, female voice, letters with their name starting with a vowel, peripheral visual location, and darker-than-background font) had an independent probability of 0.2. Therefore, a stimulus could contain 0–3 target features, although only one feature was relevant to the task at hand. This allowed us to present similar stimulus blocks during all tasks. The auditory and visual stimuli often overlapped partly in time, but a total overlap was very improbable. The target features of auditory and visual stimulus sequences were randomized independently and thus it was possible that also auditory and visual targets overlapped. In cases where two targets would be presented virtually simultaneously, the participants were instructed to press the response button twice.

### Analysis of the behavioral data

In order to minimize effects due to response selection (i.e., due to a response selection bottleneck; Pashler, 1994), responses to auditory and visual targets were given with the same button. Targets were considered in temporal order. The first response occurring within 200–1000 ms from target onset was labeled as a hit. Each response was classified only once. Hit rate (HR) was defined as the number of hits divided by the number of targets. False alarm rate (FaR), in turn, was defined as the number responses given outside the hit response window divided by the overall number of responses.

To compare task performance between single and dual task conditions, mean RTs to auditory targets were calculated for each participant across the dual tasks including the A_Phon_ task (A_Phon_V_Phon_, A_Phon_V_Spat_, and A_Phon_V_Simp_), across the dual tasks including the A_Spat_ task (A_Spat_V_Phon_, A_Spat_V_Spat_, and A_Spat_V_Simp_) and across the dual tasks including the A_Simp_ task (A_Simp_V_Phon_, A_Simp_V_Spat_, and A_Simp_V_Simp_). These mean RTs were then compared with the RT for the corresponding auditory component task performed as a single task in our previous study (Salo et al., 2013). Similarly, mean RTs to visual targets were calculated across the dual tasks including the V_Phon_ task (A_Phon_V_Phon_, A_Spat_V_Phon_, A_Simp_V_Phon_), across the dual tasks including the V_Spat_ task (A_Phon_V_Spat_, A_Spat_V_Spat_, A_Simp_V_Spat_) and across the dual tasks including the V_Simp_ task (A_Phon_V_Simp_, A_Spat_V_Simp_, A_Simp_V_Simp_) and then compared with the RT for the corresponding visual component task performed as a single task. Similar comparisons were made for each participant's HRs and FaRs.

In the ANOVAs, the degrees of freedom were Greenhouse-Geisser corrected when needed. However, the original degrees of freedom will be reported below together with the corrected *P*-value. The reported correction term ε implicates corrections.

### fMRI data acquisition and analysis

Functional brain imaging was carried out with a 3.0 T GE Signa MRI scanner (GE Medical Systems, USA) using an eight channel head coil. The functional echo planar (EPI) images were acquired with an imaging area consisting of 31 contiguous oblique axial slices (TR 2000 ms, TE 32 ms, flip angle 90°, voxel matrix 64 × 64, field of view 22 cm, slice thickness 3.0 mm, in-plane resolution 3.4 × 3.4 × 3.0 mm). Image acquisition was independent of stimulation, that is, jittered acquisition was used.

A total of 1436 functional volumes were obtained in one 48 min session. Immediately after the functional scan, a fluid attenuated inversion recovery image using the same image slices but with a denser in-plane resolution was acquired for anatomical co-alignment (FLAIR; TR 10000 ms, TE 120 ms, voxel matrix 320 × 192, field of view 22 cm, slice thickness 3.0 mm, in-plane resolution 0.7 × 1.1 mm). High-resolution anatomical images (voxel matrix 256 × 256, slice thickness 1.0 mm, in-plane resolution 1 × 1 mm) were acquired in a preceding session (Salo et al., 2013).

The data were analyzed with FSL (4.1.0, www.fmrib.ox.ac.uk/fsl) using one general linear model (GLM) with 10 explanatory variables (nine different tasks and instruction). The first four volumes of the session were excluded from analysis. The data were motion corrected, spatially smoothed (7 mm full width half maximum), and high pass filtered (cutoff 100 s). The hemodynamic response was modeled using a gamma function (mean lag 6 s, SD 3 s) and its temporal derivative. Several contrasts were defined to compare activations during dual tasks with those during the dual task baseline. For group (mixed effects) analysis, the results of lower level analyses were transformed into a standard space (MNI152; Montreal Neurological Institute). *Z*-statistic images were thresholded using clusters determined by *Z* > 2.3 and a (corrected) cluster significance threshold of *P* < 0.05 (using Gaussian random field theory).

### Comparison of dual task and single task data

Activity increments and decrements associated with dual tasks were investigated by comparing activity during the present dual tasks with activity during the corresponding single tasks measured in our previous study (Salo et al., 2013). These comparisons (fixed effects) were conducted in the space of each participant's high resolution anatomical image followed by group analysis (mixed effects) in the MNI152 space. First we contrasted brain activity during each bimodal dual task with activations during the corresponding auditory single task. We assumed that these contrasts would reveal a combination of activations associated with dual tasking and the visual component of dual task (because the visual stimuli were ignored in the auditory single tasks). Then each dual task was contrasted with the corresponding visual single task to reveal activations associated with dual tasking and with the auditory component of the dual task. The resulting statistic images were then entered into nine conjunction analyses (using the easythresh script) to reveal significant activation enhancements (*Z* > 2.3, cluster corrected *P* < 0.05) specific to dual tasking.

## Results

### Dual task performance

**Figure 2:**
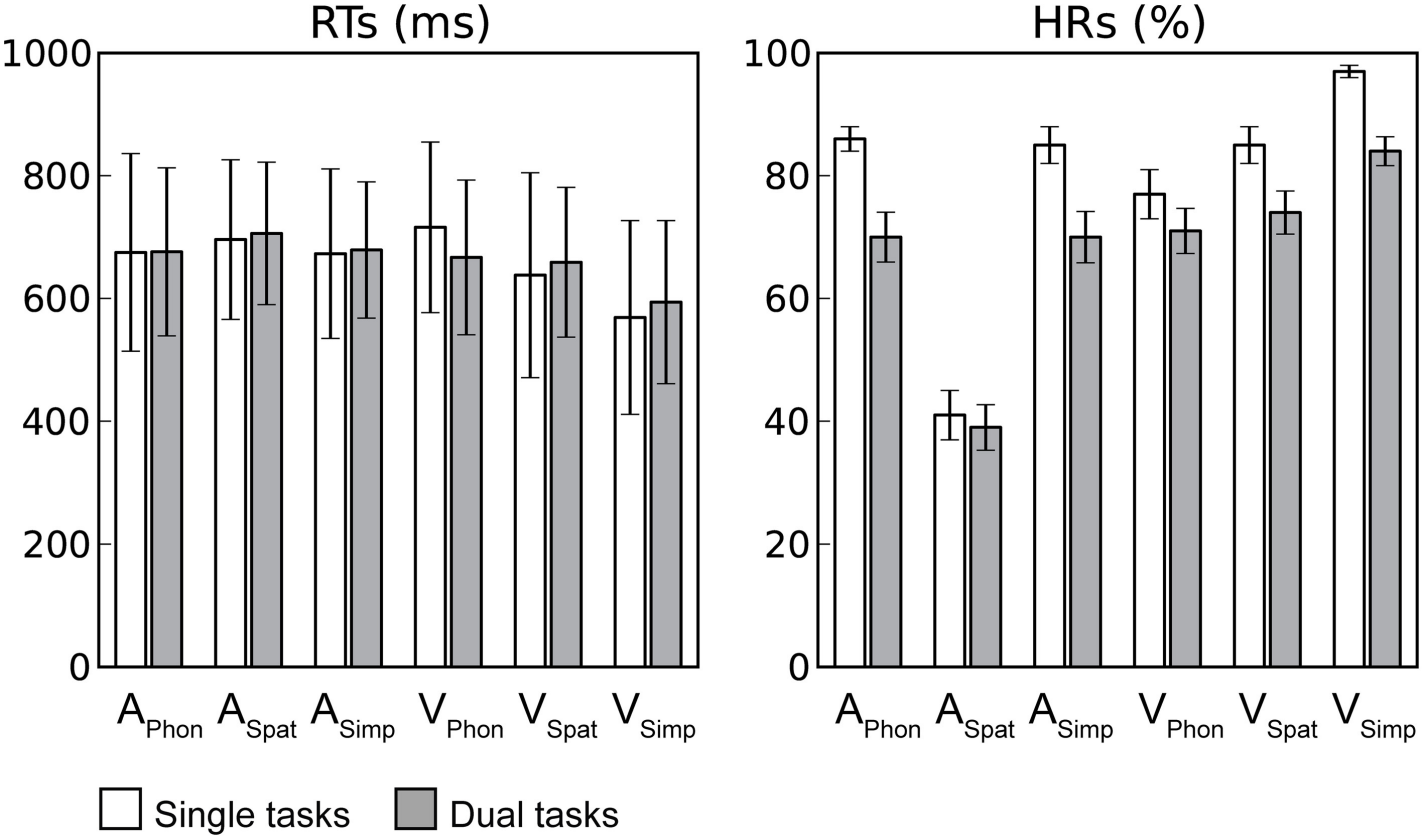
**Task performance**. Mean reaction times (RTs; includes only hit responses) and hit rates (HRs) during single (white columns) and dual (gray columns) task conditions for each auditory and visual component task. Error bars indicate SEMs (A_Phon_, A_Spat_, and A_Simp_ = Auditory phonological, spatial and simple component tasks, respectively and V_Phon_, V_Spat_, and V_Simp_ = Visual phonological, spatial and simple component tasks, respectively).

Reaction times (RTs) and hit rates (HRs) for auditory and visual targets in dual tasks were averaged across dual tasks with a particular auditory or visual component task, respectively. The RTs in the dual tasks were comparable to those in our previous study where each component task was performed as a single task by the same participants (Salo et al., 2013). For auditory RTs, a repeated-measures analysis of variance (ANOVA) with factors Condition (Single task, Dual task) and Task (Phonological, Spatial, Simple) revealed significant main effect of Task *F*_(2, 28)_ = 7.02, *P* < 0.01, the RTs being higher for A_Spat_ than for A_Phon_ or A_Simp_, but no significant effect of Condition. Likewise, a similar ANOVA for visual RTs revealed a significant main effect of Task *F*_(2, 28)_ = 103.56, *P* < 0.001, but no significant effect of Condition. However, there was a significant Condition × Task interaction *F*_(2, 28)_ = 38.53, *P* < 0.001. As seen in Figure 2 the RTs for V_Spat_ or V_Simp_ were longer during dual task conditions than during single task conditions, while the opposite was true for RTs during V_Phon_.

The HRs, in turn, were lower during the present dual tasks than during the previous single tasks clearly indicating costs of dual tasking. For auditory targets, an ANOVA with factors Condition and Task revealed significant main effects of Condition *F*_(1, 14)_ = 17.37, *P* < 0.001 and Task *F*_(2, 28)_ = 237.88, *P* < 0.001 and a significant Condition × Task interaction *F*_(2, 28)_ = 16.01, *P* < 0.001. The auditory HRs were lower during dual task conditions than during single task conditions. Within both dual and single task conditions, the HRs were lowest for A_Spat_ and similar for A_Phon_ and A_Simp_ and the HRs for A_Spat_ differed the least between the dual and single task conditions. For visual targets, a similar ANOVA revealed significant main effects of Condition *F*_(1, 14)_ = 39.94, *P* < 0.001) and Task *F*_(2, 28)_ = 21.11, *P* < 0.001 and a significant Condition × Task interaction *F*_(2, 28)_ = 4.91, *P* < 0.05. The visual HRs were lower during dual tasking than during single tasking and highest for V_Simp_ and lowest for V_Phon_, and the HR for V_Phon_ differed the least the dual and single task conditions.

The false alarm rate (FaR) was defined as the number responses given outside the hit response window divided by the overall number of responses. For each participant, the FaR in each dual task was only 7% at the highest and the mean FaR for the nine dual tasks varied between 2% (±0.5%) and 3% (±0.3%). For auditory false alarms, an ANOVA with factors Condition (Single task, Dual task) and Task (Phonological, Spatial, Simple) revealed significant main effect of Task *F*_(2, 28)_ = 11.61, *P* < 0.01, ε = 0.57 and a significant Condition × Task interaction *F*_(2, 28)_ = 14.21, *P* < 0.01, ε = 0.61. The FaRs were higher for A_Spat_ component task than for A_Phon_ or A_Simp_, this effect being stronger in single task conditions than in dual task conditions.

For visual false alarms, a similar ANOVA revealed significant main effects of Condition *F*_(1, 14)_ = 40.33, *P* < 0.001 and Task *F*_(2, 28)_ = 39.21, *P* < 0.001, and a significant Condition × Task interaction *F*_(2, 28)_ = 9.25, *P* < 0.01, ε = 0.63. The visual FaRs were higher during dual task conditions than during single task conditions. In single task conditions, the FaRs were highest for V_Phon_, intermediate for V_Spat_ and lowest for V_Simp_, whereas in dual task conditions, the FaRs for V_Phon_ and V_Spat_ did not differ and the FaR for V_Simp_ remained lowest.

### Brain activity during dual tasks in relation to the A_Simp_V_Simp_ dual task

**Figure 3:**
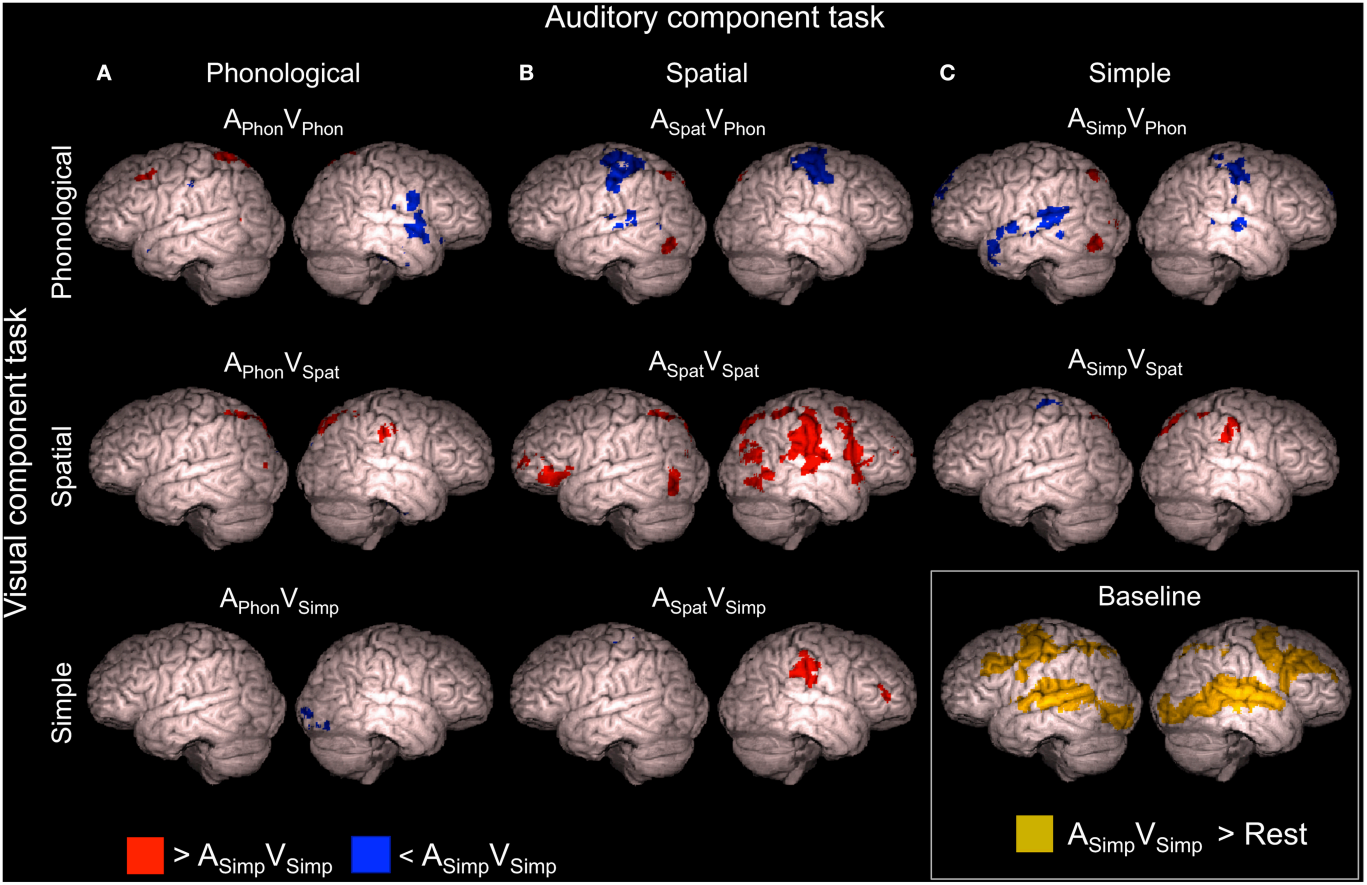
**Brain activity during dual tasks in relation to the baseline dual task**. Areas showing significant (*Z* > 2.3, cluster corrected *P* < 0.05) activity in relation to the A_Simp_V_Simp_ dual task (simultaneous speaker-gender and font-shade discrimination task) used as the baseline. Dual tasks including the **(A)** auditory phonological, **(B)** auditory spatial, and **(C)** auditory simple component tasks compared with the baseline dual task. Dual tasks including the visual phonological, visual spatial and visual simple component tasks compared with the baseline dual task are shown in top, middle and bottom rows, respectively. Brain activity during the A_Simp_V_Simp_ dual task in relation to brain activity during the resting periods is shown in the right bottom corner. Cortical activations are superimposed from 10 mm under the cortex on surface of rendered brain images.

A_Simp_V_Simp_ was used as a baseline dual task with which the other dual tasks were compared. Brain activity enhancements during this baseline dual task in relation to resting periods with no stimuli and during the other dual tasks in relation to this baseline are shown in Figure 3 (*Z* > 2.3, cluster corrected *P* < 0.05, see also Supplementary Tables 1, 2).

As seen in Figure 3, activity enhancements during the other dual tasks in comparison with the baseline dual task showed large variation. In brief, during the A_Phon_V_Phon_ dual task enhanced activity was detected in a small area of the left middle frontal gyrus (MFG; Figure 3A, top row) close to the area activated by both A_Phon_ and V_Phon_ in the single task conditions (Salo et al., 2013). The A_Spat_V_Spat_ dual task, in turn, showed enhanced activity in the right inferior parietal lobule (IPL; Figure 3B, middle row, red areas) activated by both A_Spat_ and V_Spat_ in the single task conditions. In addition, several dual tasks were associated with activity enhancements in the right IPL and in the left or bilateral superior parietal lobule (SPL; Figure 3, red areas, see also Supplementary Table 1). Activity decrements, in turn, were observed especially in dual tasks including the V_Phon_ component task in the superior temporal gyrus (STG) and pre- and postcentral gyri in one or both hemispheres depending on the auditory component task (Figure 3, top row, blue areas, see also Supplementary Table 2).

**Figure 4:**
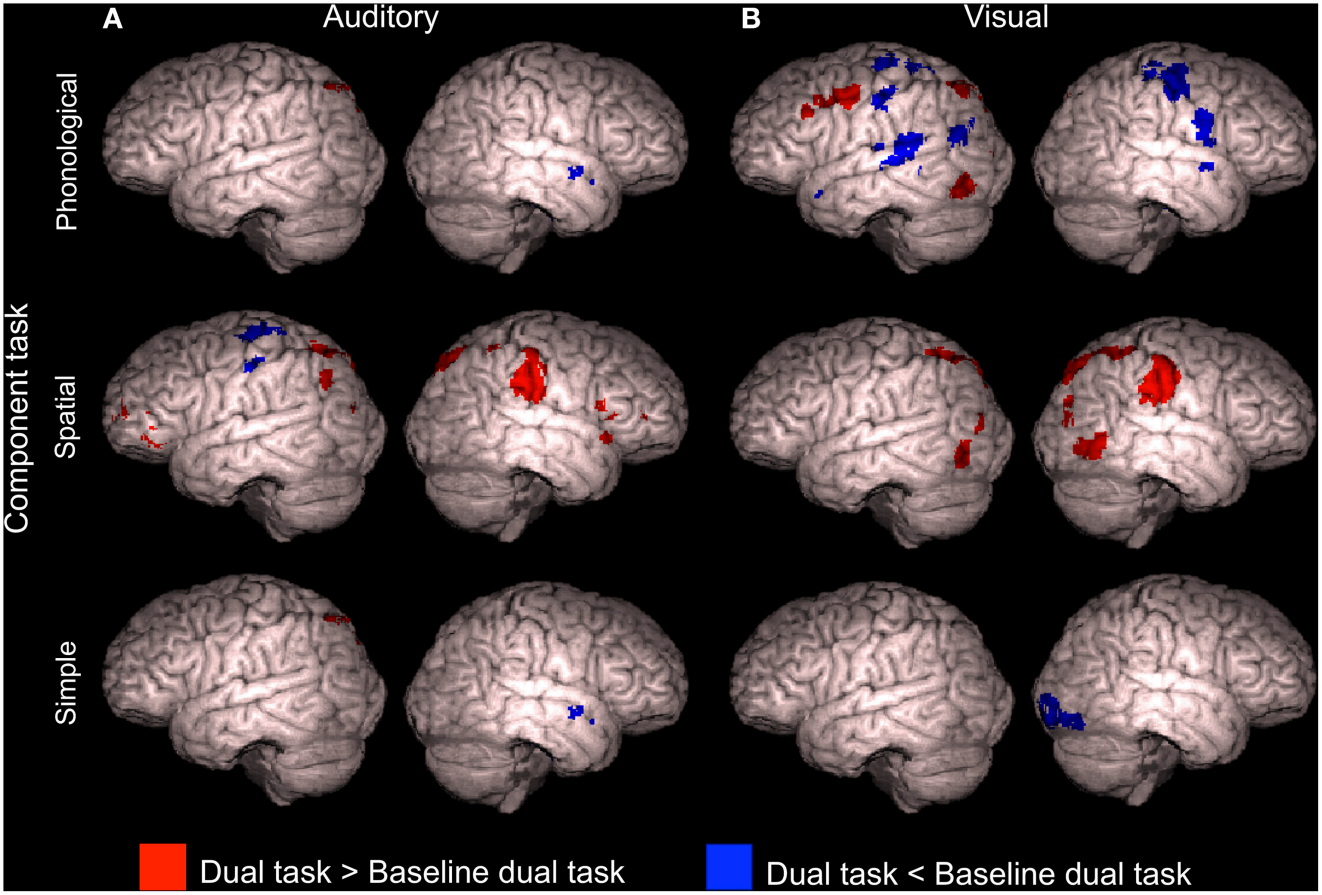
**Brain activity during dual tasks including a certain component tasks**. Areas showing significant (*Z* > 2.3, cluster corrected *P* < 0.05) activity during dual tasks including **(A)** auditory phonological (A_Phon_V_Phon_, A_Phon_V_Spat_, and A_Phon_V_Simp_), spatial (A_Spat_V_Phon_, A_Spat_V_Spat_, and A_Spat_V_Simp_) or simple (A_Simp_V_Phon_ and A_Simp_V_Spat_) component task and **(B)** dual tasks including the visual phonological (A_Phon_V_Phon_, A_Spat_V_Phon_, and A_Simp_V_Phon_), spatial (A_Phon_V_Spat_, A_Spat_V_Spat_, and A_Simp_V_Spat_) or simple (A_Phon_V_Simp_ and A_Spat_V_Simp_) component task compared with A_Simp_V_Simp_, the baseline dual task.

To reveal activity enhancements associated systematically with dual tasks including a certain component tasks, additional comparisons were implemented. Mean activations across the dual tasks including the A_Phon_ (i.e., across A_Phon_V_Phon_, A_Phon_V_Spat_ and A_Phon_V_Simp_), A_Spat_(A_Spat_V_Phon_, A_Spat_V_Spat_ and A_Spat_V_Simp_), A_Simp_(A_Simp_V_Phon_ and A_Simp_V_Spat_), V_Phon_ (A_Phon_V_Phon_, A_Spat_V_Phon_ and A_Simp_V_Phon_), V_Spat_ (A_Phon_V_Spat_, A_Spat_V_Spat_ and A_Simp_V_Spat_), and V_Simp_ (A_Phon_V_Simp_ and A_Spat_V_Simp_) component task were separately contrasted with A_Simp_V_Simp_, the baseline dual task. The results of these contrasts are shown in Figure 4. In brief, all component tasks, except V_Simp_, were associated with enhanced activity (*Z* > 2.3, cluster corrected *P* < 0.05) in the left posterior parietal cortex. Dual tasks including the A_Spat_ and V_Spat_ component tasks showed enhanced activity bilaterally in SPL and in large areas in the right IPL (Figure 4, middle row). Dual tasks including the V_Phon_ task, in turn, showed enhanced activity also in the left MFG and were associated with decreased activity bilaterally in the pre- and postcentral gyri, left SPL and IPL, left posterior STG, and in the right pars opercularis and right middle STG.

### Activity enhancements during dual tasks in relation to the component tasks

We contrasted the dual tasks with their component tasks performed separately in our previous study (Salo et al., 2013). These comparisons revealed enhanced activations not directly associated with either of the component tasks or with bimodal stimulus presentation. Thus, these activations might be specific to dual tasking. Interestingly, not all dual tasks were associated with such activity enhancements.

**Figure 5:**
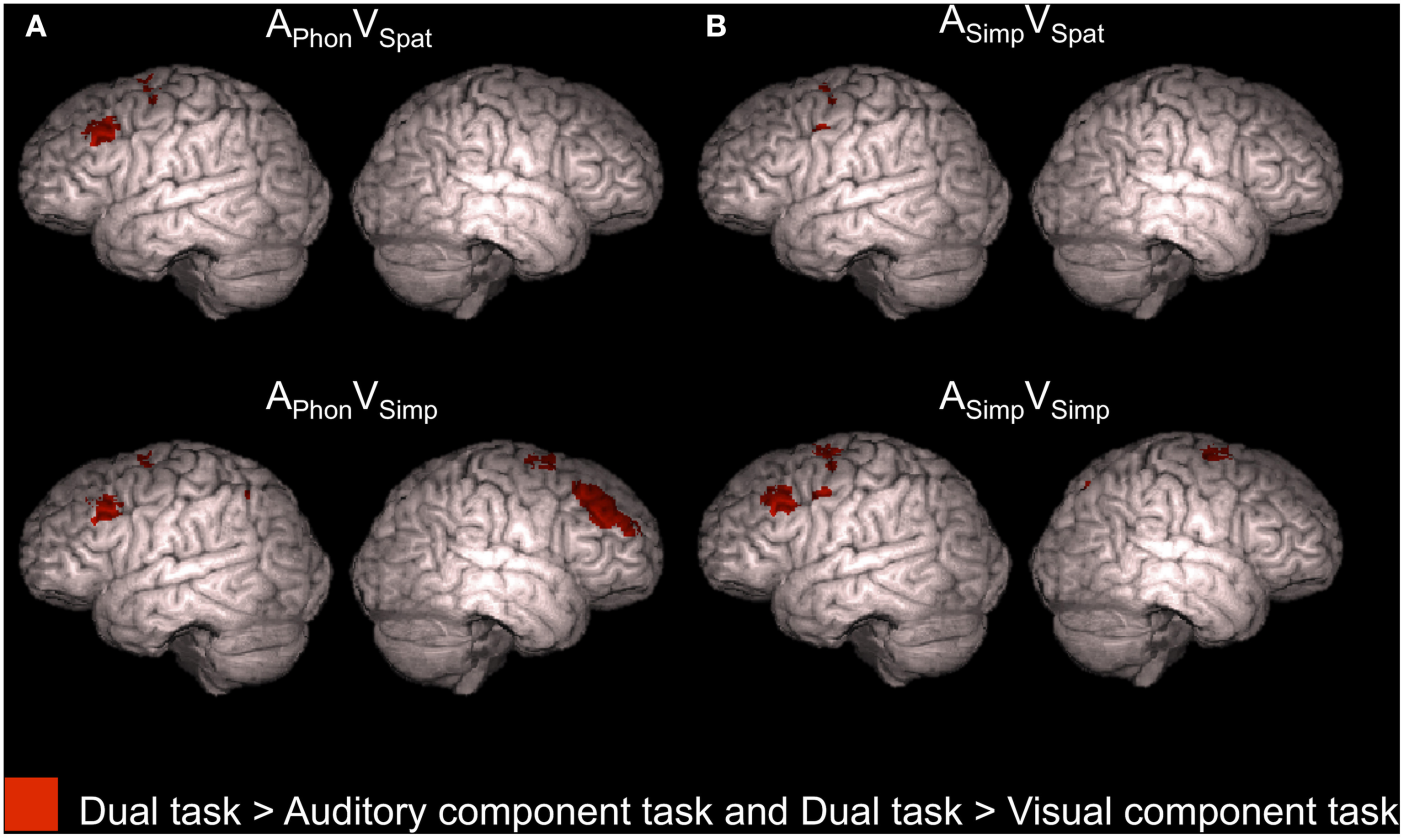
**Activity enhancements during dual tasks in relation to single tasks**. According to conjunction analyses, the colored areas showed higher activity (*Z* > 2.3, cluster corrected *P* < 0.05) for each of the four dual tasks of the present study in relation to *both* its auditory component task *and* its visual component task when performed separately in our previous study (Salo et al., 2013).

Four dual tasks, namely A_Phon_V_Spat_, A_Phon_V_Simp_, A_Simp_V_Spat_, and A_Simp_V_Simp_, each showed enhanced activity in relation to *both* its auditory component task *and* its visual component task (conjunction analysis, *Z* > 2.3, cluster corrected *P* < 0.05, see also Supplementary Table 3). As seen in Figure 5, all these dual tasks were associated with such enhanced activity in the left superior precentral gyrus (for A_Simp_V_Spat_ and A_Simp_V_Simp_ there were even two left precentral enhancement clusters). In addition, A_Phon_V_Spat_ and A_Simp_V_Simp_ showed enhanced activity in relation to both of their component tasks in the left MFG and A_Phon_V_Simp_ in the bilateral MFG. Finally, for both A_Phon_V_Simp_ and A_Simp_V_Simp_ there was such activity enhancement even in the right superior precentral gyrus.

### Activity decrements during dual tasks in relation to the component tasks

**Figure 6:**
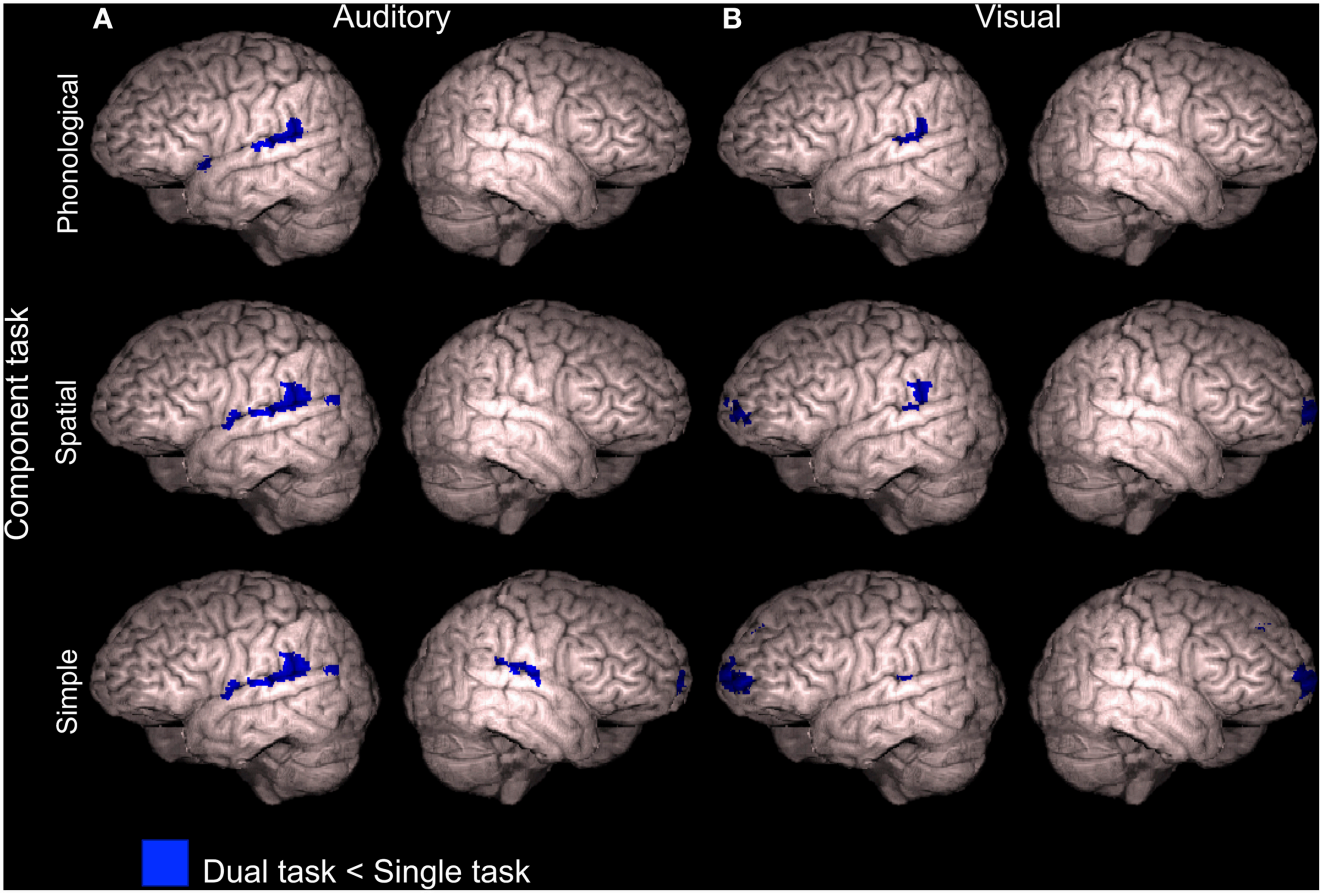
**Activity decrements during dual tasks in relation to single tasks**. Areas showing lower activity (*Z* > 2.3, cluster corrected *P* < 0.05) during dual tasks than during the component tasks performed separately in our previous study (Salo et al., 2013). **(A)** Dual tasks including the auditory phonological (A_Phon_V_Phon_, A_Phon_V_Spat_, and A_Phon_V_Simp_), spatial (A_Spat_V_Phon_, A_Spat_V_Spat_, and A_Spat_V_Simp_) or simple (A_Simp_V_Phon_, A_Simp_V_Spat_, and A_Simp_V_Simp_) component task compared with corresponding auditory component tasks (A_Phon_, A_Spat_, and A_Simp_, respectively). **(B)** Dual tasks including the visual phonological (A_Phon_V_Phon_, A_Spat_V_Phon_, and A_Simp_V_Phon_), spatial (A_Phon_V_Spat_, A_Spat_V_Spat_, and A_Simp_V_Spat_) or simple (A_Phon_V_Simp_, A_Spat_V_Simp_, and A_Simp_V_Simp_) component task compared with corresponding visual component tasks (V_Phon_, V_Spat_, and V_Simp_, respectively). Note that the brain images are tilted 20° to the left or right to reveal ventromedial brain areas.

To study activation decrements associated with dual tasking, mean activations across dual tasks including the A_Phon_ task (A_Phon_V_Phon_, A_Phon_V_Spat_, and A_Phon_V_Simp_), A_Spat_ task (A_Spat_V_Phon_, A_Spat_V_Spat_, and A_Spat_V_Simp_) and A_Simp_ task (A_Simp_V_Phon_, A_Simp_V_Spat_, and A_Simp_V_Simp_) were separately contrasted with activity during the corresponding auditory task performed as a single task in our previous study (Salo et al., 2013). As seen in Figure 6A (see also Supplementary Table 4), all these comparisons showed significantly decreased activity (*Z* > 2.3, cluster corrected *P* < 0.05) during dual tasking than during auditory single tasking in the left posterior STG. In addition, for the dual tasks including the A_Simp_ component task, activity decreased in relation to A_Simp_ performed as a single task in the right posterior STG and in the ventromedial prefrontal cortex (VMPC; Figure 6A, bottom row).

Correspondingly, dual tasks including the V_Phon_ task (A_Phon_V_Phon_, A_Spat_V_Phon_, and A_Simp_V_Phon_), V_Spat_ task (A_Phon_V_Spat_, A_Spat_V_Spat_, and A_Simp_V_Spat_) and V_Simp_ task (A_Phon_V_Simp_, A_Spat_V_Simp_, and A_Simp_V_Simp_) were contrasted with the visual component task performed as a single task. All these comparisons showed significantly lower activity (*Z* > 2.3, cluster corrected *P* < 0.05) during dual tasking than during visual single tasking in the left posterior STG (Figure 6B and Supplementary Table 4). For the dual tasks including the V_Spat_ or V_Simp_ component task, activity decreased significantly also in the VMPC (Figure 6B, middle and bottom rows).

### Task difficulty as covariate

A separate analysis was performed to investigate the possibility that task difficulty as such would explain activity observed during different dual tasks. A behavioral covariate of each participants HR was included as an additional explanatory variable in the general linear model (GLM; for other variables and details, see fMRI data acquisition and analysis). Only the A_Spat_V_Phon_ dual task showed significant activity enhancements associated with higher HRs (*Z* > 2.3, cluster corrected *P* < 0.05) and only in the anterior cingulate cortex. However, dual task effects in this model were nearly identical to those in the original analysis. Thus, variation in task difficulty did not systematically affect brain activations during dual tasking.

## Discussion

### Activity enhancements during dual tasking

We hypothesized that dual task performance is challenging especially when the two tasks require processing in the same brain areas. In particular, we assumed that supramodal phonological and spatial processing areas in the left frontal and right inferior parietal cortex (cf. Salo et al., 2013), respectively, would either show enhanced activation reflecting the double effort needed in the dual tasks where both component tasks are phonological or spatial, or decreased activation reflecting interference of simultaneous auditory and visual phonological or spatial processing. We found that in relation to A_Simp_V_Simp_ used as the baseline dual task, both dual tasks requiring overlapping processing showed specific activation enhancements not found for the other dual tasks: A_Phon_V_Phon_ was associated with enhanced activity in the left MFG (Figure 3A, top row) and A_Spat_V_Spat_ with enhanced activity in the right IPL (Figure 3B, middle row). These results appear to support the idea that the left frontal phonological areas are the bottleneck for two simultaneous phonological tasks and the right parietal spatial areas are the bottleneck for two simultaneous spatial tasks, and that activity in these bottleneck areas is enhanced when they are recruited by parallel phonological or spatial tasks, respectively.

However, when activations during A_Phon_V_Phon_ and A_Spat_V_Spat_ were compared with activations during their component tasks, no activity enhancements were found for A_Phon_V_Phon_ in the left frontal cortex or for A_Spat_V_Spat_ in the right inferior parietal cortex. These results suggest that enhanced left MFG activity during A_Phon_V_Phon_ and enhanced right IPL activity during A_Spat_V_Spat_ in relation to A_Simp_V_Simp_ were simply due to more intensive phonological processing during A_Phon_V_Phon_ and more intensive spatial processing during A_Spat_V_Spat_ than during A_Simp_V_Simp_ where the component tasks were nonphonological and nonspatial.

Additional comparisons investigating activity associated with dual tasks including a certain component task revealed that all dual tasks, except those including the V_Simp_ component task, were associated with enhanced activity in the left superior parietal cortex (Figure 4). Such activations might be explained by processing of the spatially varying auditory and visual stimuli, or by dual tasking in general, since SPL activity is also implicated in cross-modal shifting of attention (Corbetta and Shulman, 2002; Shomstein and Yantis, 2004; Corbetta et al., 2008; Salmi et al., 2009).

The comparisons between dual tasks and their component tasks performed separately as single tasks revealed no activity enhancements that were common for all nine dual tasks. Thus, the present results do not support the assumption that all dual tasks rely on some specific cortical areas. However, based on the present results, it is also clear that not all dual tasks are alike and that activations during a particular dual task depend on the task combination.

Previous studies have shown enhanced activity in the dorsolateral prefrontal cortex during dual tasking (Corbetta and Shulman, 2002; Schubert and Szameitat, 2003; Johnson and Zatorre, 2006; Stelzel et al., 2006; Johnson et al., 2007). Consistently, in the present study, we detected activity enhancements in the dorsolateral prefrontal cortex associated with dual tasking during four dual tasks (Figure 5). However, even with a more lenient threshold (*Z* > 1.6, nonsignificant), we found no such frontal activations when the dual task included either the A_Spat_ or V_Phon_ component task, or both. According to Baddeley and Hitch (1974), the A_Spat_ task and the V_Phon_ task require *mental modality change*. If prefrontal activity is related to integration of two parallel tasks (Johnson and Zatorre, 2006), then the lack of prefrontal activity enhancements in relation to single tasking during dual tasks including the A_Spat_ or V_Phon_ task, or both, suggests that mental modality change required by these tasks complicated such integration. The complexity and bimodal nature of the modality atypical A_Spat_ and V_Phon_ tasks might have made them highly demanding even during single tasking, since only in these tasks, the hit rates did not markedly decrease during dual tasking in relation to single tasking (see Figure 2).

Activation in the superior parietal cortex has been associated with cross-modal and within-modality attention shifts (Corbetta et al., 1993; Bushara et al., 1999; Weeks et al., 1999; Yantis et al., 2002; Giesbrecht et al., 2003; Shomstein and Yantis, 2004, 2006; Salmi et al., 2007, 2009), as well as with goal-oriented attention (Corbetta et al., 2008). Although all present dual tasks presumably required vigorous cross-modal shifting of attention between the auditory and visual tasks, we found no systematic parietal activity enhancements during dual tasks, when compared with the component tasks performed as single tasks (Figure 5). Perhaps spatial variation of stimuli in both modalities required shifting of spatial attention in every task and therefore parietal areas were activated already in all single tasks, resulting in weak or no parietal activation differences between the single and dual tasks.

Four dual tasks showed enhanced activation in the left superior precentral gyrus. It is probable, that this activation is related to motor responses. In both single and dual conditions, the participants were instructed to respond with their right hand index finger to targets. In the dual task conditions, the participants were required to attend both modalities simultaneously, and thus the target amount was double compared to the single task conditions.

### Activity decrements during dual tasking

The present dual tasks showed decrements of activity in relation to their component tasks when performed separately in our previous study (Salo et al., 2013). In relation to auditory component tasks, such decrements were detected mainly in the left or bilateral STG (Figure 6A). These decrements may have mainly resulted from stronger auditory attention effects during single tasks requiring selective attention to auditory modality than during dual tasks requiring division of attention between two sensory modalities. However, posterior portions of these decrements in the left hemisphere might be related to active suppression of preattentive phonological change detection in these areas (cf. Alho et al., 1998; Celsis et al., 1999) during all present dual task conditions, since left posterior STG/IPL areas showed decreased activity during dual tasking even in relation to the visual single task conditions with task irrelevant varying spoken syllables in the background (Figure 6B).

We also found that activity associated with dual tasks including the A_Simp_, V_Spat_, or V_Simp_ component task decreased in VMPC during dual tasks. VMPC has been suggested to be involved in suppressing the processing of irrelevant stimuli. This is supported by enhanced activity in VMPC and adjacent areas in response to distracting stimuli (Corbetta and Shulman, 2002; Shomstein and Yantis, 2004; Corbetta et al., 2008; Salmi et al., 2009) and enhanced electrophysiological responses to such distractors in patients with lesions in these areas (Rule et al., 2002). While in single task conditions of our previous study (Salo et al., 2013) there was probably a need to suppress the processing of stimuli in the unattended modality (see also Mittag et al., 2013), in the present dual task conditions active cross-modal suppression would have deteriorated dual task performance. Perhaps therefore there was less suppression related activity in the VMPC and adjacent areas during some dual tasks than during single tasks.

## Conclusions

The present results suggest that dual tasks including two phonological tasks (A_Phon_V_Phon_) or two spatial tasks (A_Spat_V_Spat_) are associated with specific activity enhancements in the left frontal cortex (supramodal phonological processing) and in the right inferior parietal cortex (supramodal spatial processing), respectively. Moreover, in congruence with previous studies, we observed that dual tasking with modality typical component tasks is associated with enhanced frontal activity. However, we found no such frontal activity enhancements during dual tasks including a modality atypical task (A_Spat_ or V_Phon_) and unlike for the modality typical tasks, the hit rates for the modality atypical tasks did not differ markedly between the dual and single task conditions. These results suggest that (single) tasks requiring mental modality change might be as bimodal as audio-visual dual tasks resulting in similar activations in these conditions. Taken together, our results show that all dual tasks do not simply activate the same cortical areas, but task related activations during dual tasking depend on the combination and nature of the component tasks.

### Conflict of interest statement

The authors declare that the research was conducted in the absence of any commercial or financial relationships that could be construed as a potential conflict of interest.

## References

[B1] Alais D. Morrone C. Burr D. (2006). Separate attentional resources for vision and audition. Proc. R. Soc. B. 273, 1339–1345. 10.1098/rspb.2005.3420 16777721 PMC1560294

[B2] Alho K. Connolly J. F. Cheour M. Lehtokoski A. Huotilainen M. Virtanen J.. (1998). Hemispheric lateralization in preattentive processing of speech sounds. Neurosci. Lett. 258, 9–12. 10.1016/S0304-3940(98)00836-2 9876039

[B3] Alvarez J. A. Emory E. (2006). Executive function and the frontal lobes: a meta-analytic review. Neuropsychol. Rev. 16, 17–42. 10.1007/s11065-006-9002-x 16794878

[B4] Baddeley A. D. Hitch G. (1974). Working memory, in The Psychology of Learning and Motivation: Advances in Research and Theory, ed Bower G. H. (New York, NY: Academic Press), 47–90.

[B5] Bushara K. O. Weeks R. A. Ishii K. Catalan M. J. Tian B. Rauschecker J. P.. (1999). Modality-specific frontal and parietal areas for auditory and visual spatial localization in humans. Nat. Neurosci. 2, 759–766. 10.1038/11239 10412067

[B6] Celsis P. Boulanouar K. Doyon B. Ranjeva J. P. Berry I. Nespoulous J. L.. (1999). Differential fMRI responses in the left posterior superior temporal gyrus and left supramarginal gyrus to habituation and change detection in syllables and tones. Neuroimage 9, 135–144. 10.1006/nimg.1998.0389 9918735

[B7] Corbetta M. Miezin F. M. Shulman G. L. Petersen S. E. (1993). A PET study of visuospatial attention. J. Neurosci. 13, 1202–1226. 8441008 10.1523/JNEUROSCI.13-03-01202.1993 PMC6576604

[B8] Corbetta M. Patel G. Shulman G. L. (2008). The reorienting system of the human brain: from environment to theory of mind. Neuron 58, 306–324. 10.1016/j.neuron.2008.04.017 18466742 PMC2441869

[B9] Corbetta M. Shulman G. L. (2002). Control of goal-directed and stimulus-driven attention in the brain. Nat. Rev. Neurosci. 3, 201–215. 10.1038/nrn755 11994752

[B10] Giesbrecht B. Woldorff M. G. Song A. W. Mangun G. R. (2003). Neural mechanisms of top-down control during spatial and feature attention. Neuroimage 19, 496–512. 10.1016/S1053-8119(03)00162-9 12880783

[B11] Johnson J. A. Strafella A. P. Zatorre R. J. (2007). The role of the dorsolateral prefrontal cortex in bimodal divided attention: two transcranial magnetic stimulation studies. J. Cogn. Neurosci. 19, 907–920. 10.1162/jocn.2007.19.6.907 17536962

[B12] Johnson J. A. Zatorre R. J. (2006). Neural substrates for dividing and focusing attention between simultaneous auditory and visual events. Neuromage 31, 1673–1681. 10.1016/j.neuroimage.2006.02.026 16616520

[B13] Mittag M. Inauri K. Huovilainen T. Leminen M. Salo E. Rinne T.. (2013). Attention effects on the processing of task-relevant and task-irrelevant speech sounds and letters. Front. Neurosci. 231, 1–15. 10.3389/fnins.2013.00231 24348324 PMC3847663

[B14] Mowbray G. H. (1953). Simultaneous vision and audition—the comprehension of prose passages with varying levels of difficulty. J. Exp. Psychol. 46, 365–372. 10.1037/h0054574 13109141

[B15] Pashler H. (1994). Dual-task interference in simple tasks—data and theory. Psychol. Bull. 116, 220–244. 10.1037/0033-2909.116.2.220 7972591

[B16] Roland P. E. Zilles K. (1998). Structural divisions and functional fields in the human cerebral cortex. Brain Res. Rev. 26, 87–105. 10.1016/S0165-0173(97)00058-1 9651489

[B17] Rule R. R. Shimamura A. P. Knight R. T. (2002). Orbitofrontal cortex and dynamic filtering of emotional stimuli. Cogn. Affect. Behav. Neurosci. 2, 264–270. 10.3758/CABN.2.3.264 12775190

[B18] Salmi J. Rinne T. Degerman A. Salonen O. Alho K. (2007). Orienting and maintenance of spatial attention in audition and vision: multimodal and modality-specific brain activations. Brain Struct. Funct. 212, 181–194. 10.1007/s00429-007-0152-2 17717689

[B19] Salmi J. Rinne T. Koistinen S. Salonen O. Alho K. (2009). Brain networks of bottom-up triggered and top-down controlled shifting of auditory attention. Brain Res. 1286, 155–164. 10.1016/j.brainres.2009.06.083 19577551

[B20] Salo E. Rinne T. Salonen O. Alho K. (2013). Brain activity during auditory and visual phonological, spatial and simple discrimination tasks. Brain Res. 1496, 55–69. 10.1016/j.brainres.2012.12.013 23261663

[B21] Schubert T. Szameitat A. J. (2003). Functional neuroanatomy of interference in overlapping dual tasks: an fMRI study. Cogn. Brain Res. 17, 733–746. 10.1016/S0926-6410(03)00198-8 14561459

[B22] Shomstein S. Yantis S. (2004). Control of attention shifts between vision and audition in human cortex. J. Neurosci. 24, 10702–10706. 10.1523/JNEUROSCI.2939-04.2004 15564587 PMC6730120

[B23] Shomstein S. Yantis S. (2006). Parietal cortex mediates voluntary control of spatial and nonspatial auditory attention. J. Neurosci. 26, 435–439. 10.1523/JNEUROSCI.4408-05.2006 16407540 PMC6674402

[B24] Stelzel C. Schumacher E. H. Schubert T. D'Esposito M. (2006). The neural effect of stimulus-response modality compatibility on dual-task performance: an fMRI study. Psychol. Res. 70, 514–525. 10.1007/s00426-005-0013-7 16175414

[B25] Weeks R. A. Aziz-Sultan A. Bushara K. O. Tian B. Wessinger C. M. Dang N.. (1999). A PET study of human auditory spatial processing. Neurosci. Lett. 262, 155–158. 10.1016/S0304-3940(99)00062-2 10218879

[B26] Welford A. T. (1952). The ‘psychological refractory period’ and the timing of high-speed performance—a review and a theory. Br. J. Psychol. Gen. Sect. 43, 2–19 10.1111/j.2044-8295.1952.tb00322.x

[B27] Yantis S. Schwarzbach J. Serences J. T. Carlson R. L. Steinmetz M. A. Pekar J. J.. (2002). Transient neural activity in human parietal cortex during spatial attention shifts. Nat. Neurosci. 5, 995–1002. 10.1038/nn921 12219097

