# Supplementary material for: Brain activations during bimodal dual tasks depend on the nature and combination of component tasks
Source: Front Hum Neurosci. 2015 Feb 26;9:102. doi: 10.3389/fnhum.2015.00102 (PMC4341542; doi:10.3389/fnhum.2015.00102)
Supplement: Supplementary file 1 [file Table1.PDF]

Supplementary Table 1.
Activity enhancements during dual tasks in relation to the baseline dual task.
Z-scores and MNI coordinates for global and local maxima in clusters of significant activity enhancements ($Z > 2.3$, cluster corrected $P < 0.05$) during other dual tasks in relation to $A_{Simp}V_{Simp}$, the baseline dual task. The minimum distance between maximas is 21 mm. The global maxima for each cluster is reported in bold font.

			MNI coordinates		
Hemisphere	Brain region	*Z*-score	*x*	*y*	*z*
**$A_{Phon}V_{Phon}$ > $A_{Simp}V_{Simp}$**					
**Left**	**Inferior lateral occipital cortex**	**4.92**	**-46**	**-64**	**-10**
Left	Superior lateral occipital cortex	4.50	-20	-68	52
Left	Superior lateral occipital cortex	3.85	-30	-78	22
Left	Superior lateral occipital cortex	3.37	-16	-88	46
Left	Intracalcarine cortex	2.83	-12	-78	6
**Left**	**Precentral gyrus**	**4.56**	**-42**	**0**	**28**
Left	Precentral gyrus	3.37	-50	-2	56
Left	Middle frontal gyrus	3.21	-46	26	36
**$A_{Phon}V_{Spat}$ > $A_{Simp}V_{Simp}$**					
**Left**	**Superior lateral occipital cortex**	**4.58**	**-14**	**-66**	**56**
Right	Superior lateral occipital cortex	4.42	20	-76	58
Left	Superior lateral occipital cortex	3.88	-26	-76	26
Right	Anterior supramarginal gyrus	3.63	58	-22	40
Right	Superior parietal lobule	3.47	34	-40	46
Left	Superior parietal lobule	3.01	-38	-48	56
**$A_{Phon}V_{Simp}$ > $A_{Simp}V_{Simp}$**					
–					
**$A_{Spat}V_{Phon}$ > $A_{Simp}V_{Simp}$**					
**Left**	**Superior lateral occipital cortex**	**4.04**	**-24**	**-58**	**42**
Left	Superior lateral occipital cortex	3.90	-28	-78	26
Left	Superior lateral occipital cortex	3.60	-20	-72	58
Left	Precuneus cortex	3.23	-2	-78	48
**Left**	**Inferior lateral occipital cortex**	**3.84**	**-46**	**-72**	**-8**
Left	Posterior inferior temporal gyrus	3.18	-48	-42	-20
**$A_{Spat}V_{Spat}$ > $A_{Simp}V_{Simp}$**					
**Right**	**Anterior supramarginal gyrus**	**4.82**	**60**	**-24**	**28**
Right	Superior lateral occipital cortex	4.68	22	-64	56

Left	Superior lateral occipital cortex	4.62	-26	-78	28
Left	Precuneus cortex	4.55	-6	-54	48
Right	Posterior inferior temporal gyrus	4.33	46	-54	-6
Left	Superior lateral occipital cortex	4.28	-12	-76	48
**Right**	**Insular Cortex**	**4.16**	**36**	**24**	**-2**
Left	Insular Cortex	4.00	-36	18	-2
Left	Frontal pole	3.73	-50	40	-8
Right	Precentral gyrus	3.69	52	10	36
Left	Paracingulate gyrus	3.67	-4	24	42
Left	Frontal pole	3.60	-26	60	0
$A_{Spat}V_{Simp} > A_{Simp}V_{Simp}$					
**Right**	**Anterior supramarginal gyrus**	**3.78**	**56**	**-30**	**32**
Right	Anterior supramarginal gyrus	2.72	62	-26	56
Right	Postcentral gyrus	2.69	32	-34	46
**Right**	**Insular cortex**	**4.00**	**36**	**18**	**-2**
Right	Inferior frontal gyrus	3.34	48	40	2
Right	Middle frontal gyrus	2.41	42	26	24
**Left**	**Precuneous cortex**	**3.45**	**-8**	**-48**	**54**
Right	Postcentral gyrus	3.17	16	-44	56
	**Paraginculate gyrus**	**4.07**	**0**	**30**	**38**
$A_{Simp}V_{Phon} > A_{Simp}V_{Simp}$					
**Left**	**Inferior lateral occipital cortex**	**5.28**	**-46**	**-66**	**-8**
Left	Superior lateral occipital cortex	4.44	-26	-74	24
Left	Superior lateral occipital cortex	3.93	-20	-58	46
Left	Crus I	3.23	-36	-60	-30
Left	Intracalcarine cortex	2.68	-20	-74	2
$A_{Simp}V_{Spat} > A_{Simp}V_{Simp}$					
**Left**	**Superior lateral occipital cortex**	**4.62**	**-16**	**-70**	**58**
Right	Superior lateral occipital cortex	4.55	22	-68	58
Right	Postcentral gyrus	3.52	60	-20	46
Right	Superior parietal lobule	3.48	34	-40	46
Left	Superior lateral occipital cortex	3.46	-26	-78	24
Left	Inferior lateral occipital cortex	2.56	-40	-78	8
