# Supplementary material for: Brain activations during bimodal dual tasks depend on the nature and combination of component tasks
Source: Front Hum Neurosci. 2015 Feb 26;9:102. doi: 10.3389/fnhum.2015.00102 (PMC4341542; doi:10.3389/fnhum.2015.00102)
Supplement: Supplementary file 2 [file Table2.PDF]

Supplementary Table 2.

Activity decrements during dual tasks in relation to the baseline dual task.

Z-scores and MNI coordinates for global and local maxima in clusters of significant activity decrements ($Z > 2.3$, cluster corrected $P < 0.05$) during other dual tasks in relation to $A_{Simp}V_{Simp}$, the baseline dual task. The minimum distance between maximas is 21 mm. The global maxima for each cluster is reported in bold font.

Hemisphere	Brain region	*Z*-score	MNI coordinates *x*	*y*	*z*
**$A_{Phon}V_{Phon} < A_{Simp}V_{Simp}$**					
**Right**	**Hippocampus**	**3.98**	**22**	**-16**	**-22**
Right	Posterior parahippocampal gyrus	3.60	34	-32	-14
Right	Anterior middle temporal gyrus	3.48	56	-6	-20
Right	Temporal pole	3.16	46	14	-30
Right	Postcentral gyrus	3.12	48	-10	28
Right	Cerebral white matter	3.08	24	-36	28
**Left**	**Anterior parahippocampal gyrus**	**3.64**	**-20**	**-22**	**-20**
Left	Insular cortex	3.53	-40	-8	6
Left	Precentral gyrus	3.49	-32	-20	40
Left	Temporal pole	3.48	-34	2	-16
Left	Middle frontal gyrus	3.28	-46	10	-32
Left	Postcentral gyrus	2.86	-56	-22	44
**$A_{Phon}V_{Spat} < A_{Simp}V_{Simp}$**					
**Right**	**Posterior temporal fusiform cortex**	**3.61**	**44**	**-16**	**-16**
Right	Hippocampus	3.08	20	-14	-22
Right	Cerebral white matter	2.98	22	24	4
Right	Putamen	2.80	24	6	-10
Right	Posterior inferior temporal gyrus	2.60	52	-12	-42
Right	Hippocampus	2.58	30	-40	0
**Right**	**Cuneal cortex**	**4.14**	**8**	**-86**	**24**
Right	Lingual gyrus	3.51	16	-62	-4
Left	Lingual gyrus	2.74	-10	-66	0
Right	Precuneus cortex	2.53	2	-58	22
**Left**	**Hippocampus**	**3.55**	**-18**	**-12**	**-22**
Left	Putamen	3.23	-22	4	-8
Left	Posterior inferior temporal gyrus	3.16	-44	-12	-38
Left	Posterior temporal fusiform cortex	2.67	-36	-26	-24
**$A_{Phon}V_{Simp} < A_{Simp}V_{Simp}$**					
**Right**	**Occipital pole**	**3.88**	**32**	**-90**	**2**
Right	Intracalcarine cortex	3.46	14	-84	16

Left	Cuneal cortex	2.79	-10	-88	22
Right	Precuneus cortex	2.74	6	-64	16
Right	Inferior lateral occipital cortex	2.50	42	-64	10
**$A_{Spat}V_{Phon} < A_{Simp}V_{Simp}$**					
**Left**	**Precentral gyrus**	**4.83**	**-34**	**-22**	**44**
Left	Hippocampus	4.13	-20	-20	-20
Left	Precentral gyrus	3.97	-2	-16	54
Left	Central opercular cortex	3.71	-42	-8	22
Left	Postcentral gyrus	3.63	-50	-32	56
Left	Posterior superior temporal gyrus	3.53	-62	-26	4
**Right**	**Precentral gyrus**	**3.86**	**44**	**-10**	**58**
Right	Precentral gyrus	2.46	64	4	42
**Right**	**Hippocampus**	**3.79**	**20**	**-10**	**-18**
Right	Posterior temporal fusiform cortex	3.17	38	-18	-26
Right	Putamen	3.10	26	-6	2
Right	Precentral gyrus	3.01	30	8	-32
Right	Posterior middle temporal gyrus	2.43	60	-10	-28
**$A_{Spat}V_{Spat} < A_{Simp}V_{Simp}$**					
**Left**	**Postcentral gyrus**	**3.49**	**-36**	**-26**	**48**
Left	Cerebral white matter	3.39	-20	8	24
Left	Cerebral white matter	3.31	-24	-22	28
Left	Precentral gyrus	2.73	-44	-8	26
**$A_{Spat}V_{Simp} < A_{Simp}V_{Simp}$**					
**Left**	**Postcentral gyrus**	**3.37**	**-40**	**-24**	**58**
Left	Precentral gyrus	3.14	-40	-12	34
**$A_{Simp}V_{Phon} < A_{Simp}V_{Simp}$**					
**Right**	**Precentral gyrus**	**3.75**	**46**	**-14**	**52**
	Precentral gyrus	3.59	0	-22	60
Right	Central opercular cortex	3.19	42	-4	18
Right	Posterior superior temporal gyrus	3.12	64	-16	2
Right	Postcentral gyrus	2.97	56	-18	30
Right	Precentral gyrus	2.72	32	-22	66
**Left**	**Postcentral gyrus**	**3.40**	**-34**	**-20**	**38**
Left	Posterior superior temporal gyrus	3.36	-60	-28	4
Left	Temporal pole	3.19	-54	14	-22
Left	Insular cortex	3.11	-38	0	-16
Left	Insular cortex	2.96	-40	-4	6
Left	Postcentral gyrus	2.91	-54	-26	46
**Left**	**Frontal pole**	**3.29**	**-2**	**60**	**16**
Left	Middle frontal gyrus	2.85	-28	20	42

Left	Frontal pole	2.80	-8	60	38
**$A_{Simp}V_{Spat} < A_{Simp}V_{Simp}$**					
**Right**	**Temporal occipital fusiform cortex**	**3.89**	**24**	**-58**	**-12**
Right	Intracalcarine cortex	3.05	22	-70	12
Right	Lingual gyrus	2.35	6	-74	-6
**Left**	**Postcentral gyrus**	**3.61**	**-32**	**-26**	**48**
Left	Postcentral gyrus	2.89	-44	-10	24
Left	Postcentral gyrus	2.74	-40	-24	68
Left	Postcentral gyrus	2.60	-14	-36	70
**Left**	**Amygdala**	**3.66**	**-14**	**0**	**-22**
Left	Hippocampus	3.52	-20	-22	-18
Left	Posterior inferior temporal gyrus	2.88	-44	-14	-36
Left	Posterior superior temporal gyrus	2.80	-48	-28	-2
Left	Putamen	2.65	-28	4	-2
**Right**	**Posterior middle temporal gyrus**	**3.25**	**56**	**-14**	**-12**
Right	Posterior temporal fusiform cortex	3.04	42	-20	-30
Right	Hippocampus	2.92	18	-14	-18
Right	Amygdala	2.64	30	4	-14
