# Supplementary material for: Brain activations during bimodal dual tasks depend on the nature and combination of component tasks
Source: Front Hum Neurosci. 2015 Feb 26;9:102. doi: 10.3389/fnhum.2015.00102 (PMC4341542; doi:10.3389/fnhum.2015.00102)
Supplement: Supplementary file 3 [file Table3.PDF]

**Supplementary Table 3.**

**Activity enhancements during dual tasks in relation to the component tasks.**

*Z*-scores and MNI coordinates for global and local maxima in clusters of significant activity enhancements ($Z > 2.3$, cluster corrected $P < 0.05$) for each of the four dual tasks that showed, according to conjunction analyses, significantly enhanced activity in relation to both its auditory component task and its visual component task when performed as single tasks. The minimum distance between maximas is 21 mm. The global maxima for each cluster is reported in bold font.

| Hemisphere | Brain region | *Z*-score | MNI coordinates *x* | *y* | *z* |
|---|---|---|---|---|---|
| **Dual $A_{Phon}V_{Spat}$ > Single $A_{Phon}$ and Dual $A_{Phon}V_{Spat}$ > Single $V_{Spat}$** | | | | | |
| **Left** | **Middle frontal gyrus** | **3.41** | **-26** | **2** | **54** |
| Left | Superior frontal gyrus | 3.19 | -12 | 4 | 72 |
| **Left** | **Middle frontal gyrus** | **3.61** | **-42** | **30** | **36** |
| Left | Frontal pole | 2.58 | -28 | 54 | 10 |
| **Dual $A_{Phon}V_{Simp}$ > Single $A_{Phon}$ and Dual $A_{Phon}V_{Simp}$ > Single $V_{Simp}$** | | | | | |
| **Left** | **Precuneus cortex** | **4.18** | **-8** | **-76** | **48** |
| Left | Superior lateral occipital cortex | 3.91 | -24 | -58 | 42 |
| Left | Posterior supramarginal gyrus | 3.61 | -46 | -50 | 52 |
| Right | Precuneus cortex | 3.22 | 14 | -72 | 42 |
| **Right** | **Frontal pole** | **3.88** | **44** | **38** | **36** |
| Right | Frontal pole | 2.79 | 34 | 56 | 26 |
| **Left** | **Middle frontal gyrus** | **3.61** | **-26** | **0** | **54** |
| Left | Superior frontal gyrus | 2.64 | -12 | 4 | 74 |
| **Left** | **Middle frontal gyrus** | **3.79** | **-44** | **26** | **32** |
| Left | Frontal pole | 3.04 | -30 | 38 | 20 |
| **Right** | **Superior frontal gyrus** | **3.47** | **24** | **2** | **64** |
| Right | Precentral gyrus | 3.16 | 40 | 0 | 46 |
| **Dual $A_{Simp}V_{Spat}$ > Single $A_{Simp}$ and Dual $A_{Simp}V_{Spat}$ > Single $V_{Spat}$** | | | | | |
| **Left** | **Middle frontal gyrus** | **3.52** | **-26** | **2** | **54** |
| Left | Precentral gyrus | 3.15 | -48 | 4 | 38 |
| **Dual $A_{Simp}V_{Simp}$ > Single $A_{Simp}$ and Dual $A_{Simp}V_{Simp}$ > Single $V_{Simp}$** | | | | | |
| **Left** | **Superior lateral occipital cortex** | **4.93** | **-24** | **-58** | **42** |
| Left | Precuneus cortex | 4.44 | -10 | -74 | 48 |
| Right | Precuneus cortex | 4.39 | 12 | -62 | 50 |
| Left | Posterior supramarginal gyrus | 3.27 | -44 | -50 | 52 |
| Right | Posterior supramarginal gyrus | 3.07 | 48 | -36 | 40 |
| Right | Angular gyrus | 2.81 | 28 | -54 | 34 |

| | | | | | |
|---|---|---|---|---|---|
| **Left** | **Superior frontal gyrus** | **4.29** | **-24** | **-2** | **54** |
| Left | Middle frontal gyrus | 3.78 | -42 | 32 | 36 |
| Left | Precentral gyrus | 3.71 | -50 | 4 | 40 |
| Left | Inferior frontal gyrus | 3.23 | -38 | 14 | 24 |
| **Right** | **Superior frontal gyrus** | **3.66** | **26** | **0** | **64** |
| Right | Precentral gyrus | 3.27 | 42 | 0 | 50 |
