# Supplementary material for: Brain activations during bimodal dual tasks depend on the nature and combination of component tasks
Source: Front Hum Neurosci. 2015 Feb 26;9:102. doi: 10.3389/fnhum.2015.00102 (PMC4341542; doi:10.3389/fnhum.2015.00102)
Supplement: Supplementary file 4 [file Table4.PDF]

Supplementary Table 4.
Activity decrements during dual tasks in relation to the component tasks.
Z-scores and MNI coordinates for global and local maxima in clusters of significant activity decrements ($Z > 2.3$, cluster corrected $P < 0.05$) during dual tasks in relation to single tasks. Dual tasks including the auditory phonological (A_{Phon}), spatial (A_{Spat}) or simple (A_{Simp}) component task and dual tasks including the visual phonological (V_{Phon}), spatial (V_{Spat}) or simple (V_{Simp}) component task were each compared with the corresponding component task performed as a single task. The minimum distance between maximas is 21 mm. The global maxima for each cluster is reported in bold font.

			MNI coordinates		
Hemisphere	Brain region	*Z*-score	*x*	*y*	*z*
**Dual tasks including A_{Phon} < Single A_{Phon} task**					
**Left**	**Temporal pole**	**3.88**	**-44**	**8**	**-12**
Left	Planum temporale	3.64	-64	-26	14
Left	Planum temporale	3.50	-32	-38	14
Left	Posterior supramarginal gyrus	3.31	-60	-48	14
Left	Heschl's gyrus	3.20	-42	-18	8
Left	Putamen	2.74	-20	4	-8
**Dual tasks including A_{Spat} < Single A_{Spat} task**					
**Left**	**Insular cortex**	**4.01**	**-40**	**-18**	**10**
Left	Planum temporale	3.77	-64	-26	14
Left	Posterior supramarginal gyrus	3.75	-58	-48	14
Left	Central opercular cortex	3.59	-58	-6	4
Left	Parietal operculum cortex	3.26	-34	-42	16
Left	Temporal pole	3.10	-44	8	-14
**Dual tasks including A_{Simp} < Single A_{Simp} task**					
**Left**	**Temporal pole**	**4.66**	**-48**	**12**	**-10**
Left	Insular cortex	4.47	-40	-16	8
Left	Planum temporale	3.86	-64	-26	14
Left	Planum temporale	3.72	-42	-38	16
Left	Angular gyrus	3.38	-60	-60	20
Left	Inferior lateral occipital cortex	3.23	-34	-64	8
**Left**	**Anterior cingulate gyrus**	**4.26**	**-8**	**36**	**14**
Left	Frontal pole	4.26	-4	64	-2
Left	Superior frontal gyrus	3.67	-6	32	46
Left	Superior frontal gyrus	3.66	-4	54	30
Left	Frontal pole	3.41	18	42	30
**Right**	**Insular cortex**	**4.20**	**32**	**-6**	**4**

Right	Postcentral gyrus	4.18	66	-8	10
Right	Precentral gyrus	3.38	42	-2	44
Right	Planum polare	3.30	54	4	-4
**Left**	**Paraginculate gyrus**	**4.10**	**-16**	**-46**	**18**
Right	Anterior cingulate gyrus	3.42	2	-6	30
Left	Posterior cingulate gyrus	2.82	-6	-26	28
**Dual tasks including V_{Phon} < Single V_{Phon} task**					
**Left**	**Planum temporale**	**3.70**	**-64**	**-26**	**14**
Left	Planum polare	3.34	-60	-4	4
Left	Posterior supramarginal gyrus	3.23	-46	-46	20
Left	Insular cortex	3.22	-40	-16	8
Left	Cerebral white matter	2.53	-22	-22	18
**Left**	**Precentral gyrus**	**3.76**	**-26**	**-8**	**48**
Right	Anterior cingulate gyrus	3.18	4	2	30
Left	Posterior cingulate gyrus	3.08	-6	-30	28
Left	Cerebral white matter	3.04	-16	-46	18
Left	Caudate	2.85	-18	-4	20
**Dual tasks including V_{Spat} < Single V_{Spat} task**					
**Left**	**Frontal pole**	**3.45**	**-8**	**72**	**0**
Left	Anterior cingulate gyrus	3.23	-8	38	-2
**Left**	**Inferior lateral occipital cortex**	**3.92**	**-42**	**-64**	**6**
Left	Anterior supramarginal gyrus	3.47	-68	-30	30
Left	Insular cortex	3.21	-40	-18	10
Left	Parietal operculum cortex	3.12	-34	-42	16
**Dual tasks including V_{Simp} < Single V_{Simp} task**					
**Left**	**Anterior cingulate gyrus**	**4.71**	**-4**	**32**	**12**
Left	Superior frontal gyrus	4.30	-10	36	42
Right	Frontal pole	3.89	18	36	44
	Frontal pole	3.88	0	66	-2
Right	Paracingulate gyrus	3.43	8	42	-4
Left	Frontal pole	3.13	-10	58	16
**Left**	**Posterior cingulate gyrus**	**3.80**	**-4**	**-46**	**18**
Right	Anterior cingulate gyrus	3.34	2	-8	30
Left	Cerebral white matter	3.17	-18	-36	34
Right	Posterior cingulate gyrus	2.73	14	-30	32
**Left**	**Insular cortex**	3.99	-34	-18	-4
Left	Insular cortex	3.23	-56	-4	2
Left	Cerebral white matter	3.19	-30	-40	12
Right	Anterior cingulate gyrus	2.92	-64	-26	14
